# Mitochondrial Redox Signaling and Oxidative Stress in Kidney Diseases

**DOI:** 10.3390/biom11081144

**Published:** 2021-08-03

**Authors:** Ana Karina Aranda-Rivera, Alfredo Cruz-Gregorio, Omar Emiliano Aparicio-Trejo, José Pedraza-Chaverri

**Affiliations:** 1 Laboratorio F-315, Departamento de Biología, Facultad de Química, Universidad Nacional Autónoma de México, Mexico City 04510, Mexico; anaaranda025@gmail.com; 2 Posgrado en Ciencias Biológicas, Universidad Nacional Autónoma de México, Ciudad Universitaria, Mexico City 04510, Mexico; 3 Laboratorio F-225, Departamento de Biología, Facultad de Química, Universidad Nacional Autónoma de México, Mexico City 04510, Mexico; cruzgalfredo@gmail.com; 4 Departmento de Fisiopatología Cardio-Renal, Instituto Nacional de Cardiología “Ignacio Chávez”, Mexico City 14080, Mexico; emilianoaparicio91@gmail.com

**Keywords:** acute kidney injury (AKI), chronic kidney disease (CKD), tricarboxylic acid (TCA) cycle, mitochondrial metabolism, mitochondrial redox signaling, mitochondrial proteins, oxidative phosphorylation (OXPHOS), fatty acid (FA) β-oxidation, mitochondrial dynamics, biogenesis, mitophagy

## Abstract

Mitochondria are essential organelles in physiology and kidney diseases, because they produce cellular energy required to perform their function. During mitochondrial metabolism, reactive oxygen species (ROS) are produced. ROS function as secondary messengers, inducing redox-sensitive post-translational modifications (PTM) in proteins and activating or deactivating different cell signaling pathways. However, in kidney diseases, ROS overproduction causes oxidative stress (OS), inducing mitochondrial dysfunction and altering its metabolism and dynamics. The latter processes are closely related to changes in the cell redox-sensitive signaling pathways, causing inflammation and apoptosis cell death. Although mitochondrial metabolism, ROS production, and OS have been studied in kidney diseases, the role of redox signaling pathways in mitochondria has not been addressed. This review focuses on altering the metabolism and dynamics of mitochondria through the dysregulation of redox-sensitive signaling pathways in kidney diseases.

## 1. Introduction

Kidney diseases are a severe health problem that causes high economic costs worldwide in medical attention, emergency, therapies, among others [1,2]. These are divided into acute kidney injury (AKI) and chronic kidney diseases (CKD). AKI encompasses a set of pathologies characterized by the rapid loss of renal function in a short period [3]. AKI is often caused by the use of chemotherapeutics agents such as cisplatin, episodes of renal ischemia/reperfusion (I/R), and exposure to contaminants [4]. AKI is associated with high morbidity and mortality, contributing to CKD development and affecting approximately between 7% and 12% of the world [5]. CKD cause renal fibrosis development [6,7,8]. The latter comprises an unsatisfactory repair process and is the consequence of severe and persistent damage that does not restore organ function [9]. Renal fibrosis, in turn, is one of the principal mechanisms involved in AKI to CKD transition [5].

Mitochondria are responsible for several cell functions such as cell growth, cell survival, and apoptosis induction, playing a significant role in kidney physiology and the development of kidney diseases. Mitochondria also coordinate the biosynthesis of lipids, amino acids, and nucleotides and bioenergetics processes such as tricarboxylic acid (TCA) cycles, electron transport systems (ETSs), and fatty acids (FA) β-oxidation [10]. During these processes, reactive oxygen species (ROS) are produced. Low levels of ROS are needed to regulate cellular signaling, but an excess of ROS induces oxidative stress (OS). OS causes oxidative damage in organelles including mitochondria and biomolecules, such as proteins, lipids, and deoxyribonucleic acid (DNA), which may be conducive to cell death. Indeed, OS is associated with AKI development and its transition to CKD, where mitochondria dysfunction is the principal characteristic of both [11,12]. Although mitochondrial metabolism, ROS production, and OS have been studied in kidney diseases, the role of redox signaling pathways in renal mitochondria impairment is not well understood. This review focuses on altering the metabolism and dynamics of mitochondria through the dysregulation of redox-sensitive signaling pathways in kidney diseases.

## 2. Redox-Sensitive Signaling in Kidney Diseases

ROS at low levels act as secondary messengers, activating signaling pathways and cellular enzymes and regulating several cellular processes such as cell proliferation, survival, and growth [13]. Among the plethora of ROS, hydrogen peroxide (H_2_O_2_) and nitric oxide (^•^NO) are the central redox signaling agents [13,14,15,16]. These ROS induce oxidative post-translational modifications (Ox-PTMs) in proteins that contain redox-sensitive amino acid residues, regulating their structure, localization, and function [17,18]. These redox-sensitive amino acids are arginine (Arg), cysteine (Cys), histidine (His), lysine (Lys), methionine (Met), proline (Pro), threonine (Thr), and tyrosine (Tyr) [19,20]. However, Cys and Met are most prone to be attacked by ROS. These amino acids contain oxidizable sulfur groups on the side chains, of which the oxidation states depend on the redox microenvironment changes [13]. Thus, the protein function is regulated by the oxidate states of these amino acids [21,22].

**Figure 1:**
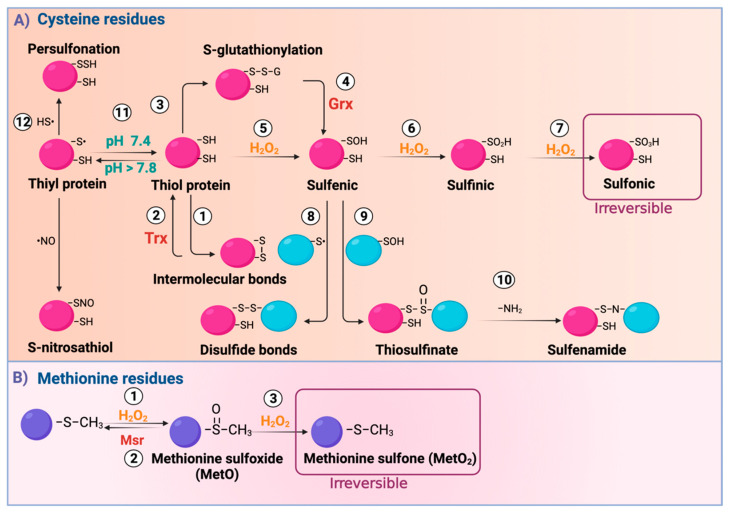
Reactive oxygen species (ROS) induce modifications in cysteine (Cys) and methionine (Met) residues. (**A**) ROS oxidize Cys residues in its thiol form, named thiol (SH) protein, forming (1) intermolecular bonds, (2) reversed by thioredoxin (Trx), or (3) S-glutathionylation (R–SSG). R–SSG can be reversed by (4) the glutaredoxin (Grx) enzyme, acquiring a sulfenic (R–SOH) form. Moreover, (5) R–SOH is formed through oxidation by H_2_O_2_ from SH. If oxidation continues, R–SOH forms (6) sulfinic (R–SO_2_H) or (7) sulfonic (R–SO_3_H); the last is irreversible. Furthermore, R–SOH can condense with another R-SOH to form (8) disulfide bonds (R–S–S–R’) or (9) thiosulfinate [R–S(O)–S-R]. The latter can react with amide groups (–NH_2_), forming (10) sulfenamide (R–SN–R′). At the alkaline cellular microenvironment (pH > 7.8), R-SH proteins are deprotonated, forming (11) thiyl proteins (–S•). The latter can react with hydrogen sulfide (H_2_S) to form (12) persulfide (RSSH) by persulfination (R–S–S–H). –S• can also react with nitrosothiol (•NO), forming S-nitrosothiol (RSNO). (**B**) Met residues are oxidized to form (1) methionine sulfoxide [MetO (R–SOCH_3_)], reversed by (2) methionine sulfoxide reductase (Msr). However, if oxidation persists, MetO is further oxidized by H_2_O_2_ to (3) methionine sulfone [MetO_2_ (RSO_2_CH_3_)], which is irreversible. H_2_O_2_: hydrogen peroxide; HS^•^: hydrosulfide radical. Created with BioRender.com.

Cys residues perform structural functions, such as the assembly of iron-sulfur (Fe-S) groups, heme prosthetic groups, and zinc finger motifs and are essential in the active sites of enzymes. The sulfur in Cys is a large, polarizable, electron-rich atom. These characteristics give it high reactivity and the ability to adopt multiple oxidation states. In addition, pKa influences the formation of the nucleophilic thiolate anion (S^−^). Because the surrounding milieu influences pKa, the thiol (SH) protonation form of Cys depends on the cellular microenvironment. For example, at physiological pH (pH 7.3), the pKa of Cys is 8.3, which means that Cys is in a protonated and less reactive state [23]. However, if the cellular microenvironment becomes alkaline (pH < 7.8), Cys can adopt a deprotonated state S^−^. Note that positively charged amino acids adjacent to Cys can substantially decrease the pKa of SH. Furthermore, the presence of other contiguous positive charges, such as the positive partial charge of a dipole, can also reduce the pKa of Cys, making them more reactive [24]. On the other hand, since Cys oxidation reactions are predominantly bimolecular nucleophilic substitution (S_N_2) reactions in the protonated form, steric hindrance can prevent Cys oxidation [25]. In this way, in the steric hindrance, the surrounding amino acids and redox microenvironment will determine whether a Cys will be reactive to undergo redox modification, dictating selectivity for SH modification. Therefore, there is a wide range of Ox-PTMs that also depend on the present electrophiles. For example, H_2_O_2_ oxidizes SH, with an oxidate state of −2, in an oxidative microenvironment, producing sulfenic acid (R–SOH) with an oxidate state of 0 [26]. R-SOH can react with proximal SH groups to form disulfide bonds (S–S), with a −1 oxidate state or s-glutathionylation (R-SSG) by reacting with glutathione (GSH) (Figure 1) [27,28]. This reaction is reversed by glutaredoxin (Grx). However, under OS, R-SOH forms sulfinic acid (R–SO_2_H) with a +2 oxidate state or sulfonic acid (R–SO_3_H) with a +4 oxidate state. The latter is not enzymatically reversible [29]. R–SOH can also be condensed with another R–SOH to form thiosulfinate [R–S(O)–S–R’]. R–S(O)–S–R’, in turn, reacts with amine or amide groups to form sulfenamide (R–SN–R’) [30]. Additionally, Cys can suffer from other modifications such as S-nitrosylation and persulfonation. Regarding S-nitrosylation, it comprises the ^•^NO covalent link to the thyil radical (R–S^•^) to form S-nitrosothiol (R–SNO), [31] and in persulfonation, the hydrogen sulfide (H_2_S) reacts with R–S^•^ to form persulfide (R–S–SH) [20].

On the other hand, Met residues are oxidated by H_2_O_2_, generating methionine sulfoxide [MetO (R–SOCH_3_)] [32]. This oxidation is reversed by methionine sulfoxide reductase (Msr). However, if OS persists, MetO is transformed into methionine sulfone [MetO_2_ (RSO_2_CH_3_)], an enzymatically irreversible product [33].

The alkalinization of the mitochondrial matrix is due to pumping protons from the matrix into the intermembrane space (IMS). It has a tremendous impact on the potential reactivity of Cys, because alkalinization induces Cys protein residues to exist in an ionized state, which gives them a higher reactivity for ROS oxidation. Thus, mitochondria harbor a unique environment that promotes Cys modification.

As mentioned, there are Ox-PTMs classified as enzymatically reversible and irreversible. Ox-PTMs are reversible by thioredoxin (Trx), peroxiredoxin (Prx), Grx, glutathione peroxidase (GPx), Msr isoform A (MsrA), or the GSH [34,35]. Since reversible Ox-PTMs have enzymatic systems that remove oxidations, they are considered part of cellular signaling processes [34]. In contrast, irreversible ROS modifications are not considered part of cell signaling, because they do not have enzymatic reduction mechanisms [18].

Regarding cysteine persulfidation, it is a reversible Ox-PTM modification of SH to RSSH, which can be formed by reacting with H_2_S, more precisely HS^−^, and oxidized protein thiols, reaction between inorganic polysulfides and protein thiolates, and radical reaction by other reactive sulfur species [24]. Persulfidation regulates fine tune protein function, localization and interaction in cells [36]. It also modulates biological processes, including autophagy, cellular metabolism, inflammation, cell cycle, and cell death under physiological and pathological contexts [37]. It has been shown that the Cys’ architecture and spatial arrangement determine if the residue can be persulfonated and the effect on protein function. Allosteric impediments protect Cys residues from being oxidized under SOH, preventing permanent damage and preserving protein function. Likewise, in a reduced environment, this Ox-PTM is reversed [38]. Additionally, persulfonation depends on ROS levels in the cells. The latter is supported, because the transient ROS increase might augment oxidized Cys forms (SOH and S–S), highly reactive to H_2_S [39]. Thus, the reversibility of this Ox-PTM relies on Cys residues and the cellular microenvironment.

Ox-PTMs inactivate numerous proteins that contain Cys groups, such as protein tyrosine phosphatases (PTPs). The inactivation of PTPs prevents the deactivation of protein tyrosine kinases (PTKs), maintaining the activation of cell signaling pathways, such as mitogen-activated protein kinases (MAPK), promoting cell proliferation [40]. In addition, ROS such as H_2_O_2_ have also been shown to promote Cys oxidation and the formation of S–S in proteins such as growth factor receptors (GFRs), inducing their activation and cell growth. Since oO-PTMs regulate cellular functions involving cell proliferation, growth, migration, differentiation, and death, the concentration of ROS must be balanced to maintain cell homeostasis and prevent kidney diseases [13].

In kidney physiology, 25% of mitochondrial Cys in proteins can suffer nitrosylation, and around 70% of these proteins depend on the activity of endothelial nitric oxide synthase (eNOS) [41]. These nitrosylations are protective in the kidneys. For instance, Zhou et al. [42] reported that in I/R, the denitrosylase enzyme aldo-keto reductase family 1 member A1 (AKR1A1) is associate with kidney damage. In contrast, its deletion is protective in this organ. The authors also found that S-nitroso-coenzyme A (CoA) reductase induces pyruvate kinase isoform M2 (PKM2) nitrosylation, inhibiting its activity. This nitrosylation favors the pentose phosphatase pathway instead of glycolysis. The latter reduces equivalents increase, attributed to ROS detoxification, which has a protector effect during I/R [42]. S-glutathionylation is also a protective Ox-PTM in renal mitochondria [43]. In the kidneys, the reduction of mitochondrial protein S-glutathionylation induced by folic acid is associated with acute kidney damage [44]. The latter has been demonstrated, because of glutathionylated proteins decrease and the levels of GSH and the activity of the enzymes involved in S-glutathionylation in the mitochondria 24 h (h) after treatment with folic acid [44]. In the cisplatin-induced AKI model, the reduction of Met is a protective Ox-PTM, since MsrA deficiency exacerbates cisplatin-induced damage by increasing mitochondrial susceptibility [45]. Furthermore, mice MsrA^−/−^ are more sensible to kidney injury induced by I/R [46]. The deficiency of MsrA, cystathionine-β-synthase (CBS), and cystathionine-γ-lyase (CSE), which are enzymes involved in the transsulfuration pathway, decreases homocysteine and H_2_S [46]. Low levels of H_2_S have also been reported after unilateral ureteral obstruction (UUO) due to CBS and CSE decrease, inducing superoxide anion radical (O_2_^•−^) and H_2_O_2_ production. The latter promotes the augment of oxidative damage markers such as malondialdehyde (MDA) and 4-hydroxynonenal (4-HNE) [47]. The MsrA deficiency in this model contributes to fibrosis development by increasing collagen deposition and augmenting fibrosis markers [48]. Therefore, MsrA regulates the Met metabolism and the production of H_2_S in the kidney [49].

Indeed, H_2_S regulates several kidney biological processes. CSE and CBS are expressed in the kidney, producing H_2_S that controls sodium reabsorption and glomerular filtration [50]. In addition, CSE is commonly expressed in endothelial and mesangial cells and podocytes [51]. In kidney diseases, H_2_S ameliorates renal damage [52]. It has been shown that H_2_S attenuates kidney injury during I/R and diabetic kidney disease (DKD). In animal models of I/R, H_2_S donors administration before or after I/R ameliorates renal damage by decreasing OS, inflammation, and apoptosis [53]. Furthermore, in patients, it has been found that CSE messenger RNA (mRNA) expression positively correlates with glomerular filtration rate recovery after two weeks of kidney transplantation [54], suggesting a protective role of H_2_S. Supporting the latter, CBS and CSE levels and H_2_S production are low in animal models suffering I/R [53]. Regarding DKD, patients with type 2 diabetes show lowed H_2_S plasma levels than non-diabetic people [55]. In UUO, the low production of H_2_S is also due to the fact that CBS levels are decreased [56]. In this model, H_2_S decrease is related to fibrosis and inflammation development. Following this, in vitro, sodium hydrosulfide (NaHS) treatment reduces fibrosis and inflammation by inhibiting transforming growth factor-beta 1 (TGFβ1) [56]. Together, these data support the relevance of H_2_S signaling in kidney injury.

Cys persulfidation is an essential process in kidney physiology and disease, protecting against kidney injury [24]. Persulfidation in Cys 105 of mitochondrial glyceraldehyde 3-phosphate dehydrogenase (GAPDH) elevates its enzymatic activity [57]. In contrast, the persulfidation in Cys 156 or Cys 152 induces enzymatic deactivation [58]. Moreover, the persulfidation in Keap 1 Cys 150 promotes nuclear factor erythroid 2–related factor 2 (Nrf2) release and translocation to the nucleus, activating its genes targets [59]. This mechanism is critical for regulating redox homeostasis in the kidney. In renal pathologies, Nrf2 is deactivated, leading to OS increase [60,61]. In the kidney, persulfides regulate blood pressure and sodium reabsorption. In line with this, in tubular epithelial cells, the persulfidation of epidermal growth factor receptor (EGFR) (Cys 797 and 798) results in the loss of function of sodium/potassium-adenosine triphosphatase (Na^+^/K^+^-ATPase) [62]. Cys persulfide is produced by CSE and CSB, which are depleted in kidney diseases. However, a previous study showed that mice lacking CSE and CBS have significant levels of Cys persulfide proteins, which suggested the possibility of alternative processes [63,64,65]. Indeed, cysteinyl-transfer RNA (tRNA) synthetases (CARSs) act as cysteine persulfide synthases in vivo [66]. Moreover, CARSs are also involved in the regulation of mitochondrial bioenergetics (oxygen consumption and membrane potential) and dynamics (dynamin-related protein 1 (Drp1)) [66]. Thus, this mechanism may be active in kidney diseases, which deserves investigation. The latter might suggest therapeutic targeting OS-induced mitochondrial dysfunction in renal pathologies.

## 3. Ox-PTMs Regulate Manganese Superoxide Dismutase (Mn-SOD) in Kidney Injury

Mn-SOD is in the mitochondrial matrix, while copper/zinc-SOD (Cu/Zn-SOD) is in the space of the inner mitochondrial membrane (IMM) and IMS. These two enzymes catalyze the dismutation of O_2_^•^^−^ to H_2_O_2_ in the mitochondrial matrix and IMS, respectively [67]. This dismutation is crucial to avoid the O_2_^•^^−^-induced ferric iron (Fe^3+^) to ferrous iron (Fe^2+^) reduction in Fe-S clusters of critical enzymes such as aconitase (Acn). The latter leads to the release of Fe^2+^ and the inactivation of these enzymes [68]. During Fenton/Haber–Weiss reaction, Fe^2+^ reacts with H_2_O_2_ to produce a highly reactive ROS, hydroxyl radical (^•^OH), so the regulation of O_2_^•−^ is essential to maintain mitochondrial homeostasis. Moreover, O_2_^•−^ overproduction, directly and indirectly, leads to the inactivation of Mn-SOD, promoting mitochondrial dysfunction. For instance, O_2_^•−^ can react with ^•^NO to produce peroxynitrite (ONOO^−^), and ONOO^−^ can induce Mn-SOD deactivation via nitration of Tyr34 residue in its active site [69].

Mn-SOD can also be S-glutathionylated in Cys 196, avoiding irreversible oxidation of SH. Renal I/R injury induces O_2_^•−^ and ONOO^−^ production [70], increasing nitration levels of mitochondrial proteins such as Mn-SOD and cytochrome c (cyt c), inactivating them and inducing OS and mitochondrial dysfunction [71,72,73,74]. Likewise, in folic-acid-induced renal damage, Mn-SOD activity reduction in isolated mitochondria is related to decreased mitochondrial S-glutathionylation [44], making it more susceptible to nitration. Moreover, in human renal transplants and experimental rat models of chronic renal nephropathy, there are elevated levels of ONOO^−^ [72].

In DKD, mitochondrial OS reduces Mn-SOD enzyme activity due to Tyr nitration of this enzyme. Interestingly, the use of resveratrol, a potent antioxidant, reduces OS and Tyr nitration of Mn-SOD, preserving Mn-SOD activity. Moreover, kidneys of mice treated with streptozotocin to induced diabetic nephropathy (DN) show nitration in Mn-SOD Tyr 34, which results in a decrease of Mn-SOD. However, antagonists of thromboxane A2 receptors reduce diabetes-induced renal injury, which is associated with Mn-SOS Tyr nitration reduction [75].

In models of hypertension-related kidney injury, where hypertension is induced by angiotensin II (Ang II), the production of O_2_^•−^ is promoted through the activation of nicotinamide adenine dinucleotide phosphate (NADPH) oxidases (NOXs) [76]. In these models, Mn-SOD activity deactivation is also associated with Tyr nitration, inducing OS [77]. Moreover, spontaneously hypertensive rats treated with N(G)-nitro-L-arginine-methyl ester (L-NAME), a potent nitric oxide synthase (NOS) inhibitor, reduce nitration of Mn-SOD, preserving its activity [78].

OS closely regulates aging-related kidney dysfunction, primarily by mitochondrial ROS (mtROS). A protein closely associated with aging is Klotho. This protein induces the activation of transcription factors such as forkhead box O (FoxO) that cause the expression of antioxidant enzymes such as Mn-SOD. Interestingly, Klotho^−/−^ mouse models show high nitrotyrosine levels in Mn-SOD [79].

## 4. Crosstalk between NOXs and Mitochondria in Kidney Diseases

Mitochondria and NOXs are the primary ROS sources in the kidney. The NOXs family consists of seven isoforms, being NOX1, NOX2, NOX4, and NOX5, the most expressed in renal cells [80]. The O_2_^•−^ production of NOX1 and NOX2 needs to assemble membrane subunit p22-phox and the cytosolic subunits p47- and p67-phox and ras-related C3 botulinum toxin substrate 1 (Rac1), while NOX4, abundantly expressed in mitochondrial membranes of renal cells, does not require cytosolic subunits and produces H_2_O_2_ [81,82,83].

**Figure 2:**
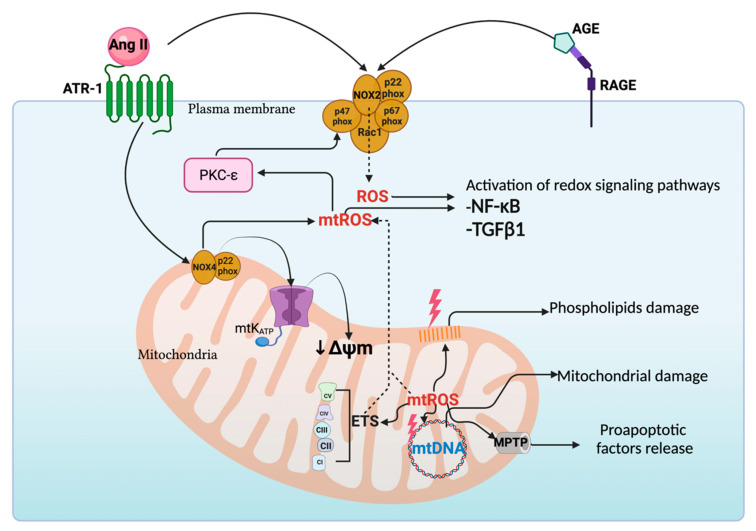
Crosstalk between nicotinamide adenine dinucleotide phosphate (NADPH) oxidases (NOXs) and mitochondria in the kidney. Angiotensin II (Ang II) binds to the angiotensin type 1 receptor (ATR-1), which activates NOX2 and NOX4. In addition, the binding of advanced glycation end products (AGEs) to the receptor for advanced glycation end products (RAGE) induces the activation of NOX2. Furthermore, protein kinase C (PKC) epsilon (PKC-ε), activated by mitochondrial ROS (mtROS), activates NOX2 through the p47-phox subunit, inducing ROS production. NOXs-induced ROS promote the phosphorylation and opening of an mitochondrial adenosine triphosphate (ATP)-sensitive potassium K channel (mt-K_ATP_), decreasing mitochondrial membrane potential depolarization (↓ΔΨm). ROS and mtROS activate the redox signaling pathways: transforming growth factor-beta 1 (TGFβ1) and nuclear factor kappa-light-chain-enhancer of activated B cells (NF-κB). mtROS also affect mitochondrial function by inducing damage to mitochondrial deoxyribonucleic acid (DNA) (mtDNA) and phospholipids that, in the last instance, generates mitochondrial dysfunction. The mtROS also favor the mitochondrial permeability transition pore (MPTP) opening, inducing the release of proapoptotic factors into the cytosol. p67-phox: subunit from NOX2; p22-phox: subunit from NOX2 and NOX4; Rac1: Ras-related C3 botulinum toxin substrate 1; ETS: electron transport system; CI: complex I; CII: complex II; CIII: complex III; CIV: complex IV; CV: complex V. Created with BioRender.com.

The levels of NOXs augment in several AKI and CKD models, inducing ROS overproduction [84]. In the folic acid model, NOXs-linked ROS overproduction (in the kidney cortex, proximal tubules (PT), and distal tubules (DT)) is related to mtROS enhancement, because these two sources establish a pathological circle of ROS production, favoring AKI to CKD transition [44]. In the 5/6 nephrectomy model, mtROS also induce NOXs activation, increasing inflammation and fibrosis. Moreover, this mechanism contributes to fibrosis development in UUO [85]. Following the latter, several authors have established that in kidney pathologies, mtROS and NOXs-produced ROS increase mitochondrial damage and mitochondrial membrane potential depolarization (↓ΔΨm) (Figure 2) [86,87]. Ang II with the angiotensin type 1 receptor (ATR-1) also participates in the crosstalk between NOXs (NOX2 and NOX4) and mitochondria [88]. In addition to Ang II, in DN, the interaction of advanced glycation end products (AGE), produced by high glucose levels, activates NOXs by AGE receptor (RAGE) to generate ROS production [89]. Protein kinase C (PKC) epsilon (PKC-ε) also activates NOXs by inducing the phosphorylation of p47-phox, triggering ROS production [90]. NOXs-induced ROS cause the opening of a mitochondrial adenosine triphosphate (ATP)-sensitive potassium (K) channel (mt-K_ATP_), triggering ↓ΔΨm [91]. In AKI and CKD models, the use of the NOXs inhibitor, apocynin, decreases mtROS production [92,93], supporting the idea that crosstalk between NOXs and mitochondria is involved.

The binding of Ang II and AGEs to their receptors activates TGFβ1 and nuclear factor kappa-light-chain-enhancer of activated B cells (NF-κB) redox-sensitive signaling pathways (Figure 2) [94]. Furthermore, mtROS can stimulate TGFβ1 through the upregulation of Smad 2/3, inducing its nuclear translocation. The latter is supported by the fact that the mitochondrial-targeting antioxidants coenzyme Q (mitoQ) and 2-(2,2,6,6-tetramethylpiperidin-1-oxyl-4-ylamino)-2-oxoethyl)triphenylphosphonium chloride (mitoTEMPO) prevents the activation of TGFβ1 along with the transcription of *TGFβ1* genes [95]. mtROS also activate NF-κB by inducing monocyte/macrophage infiltration, increasing interstitial inflammation in UUO kidneys, and treating the antioxidant curcumin to decrease them and preventing interstitial inflammation [96]. mtROS also activate NF-κB in macrophages. In this sense, Herb et al. [97] demonstrated that in macrophages, mtROS, and not ROS produced by NOX2, activate NF-κB by deactivating the regulatory subunit of inhibitor IKK complex (IKKγ) through the disulfide linkage formation. However, in kidney diseases, this mechanism has not been investigated.

TGFβ1 and NF-κB are also localized in mitochondria, regulating mitochondrial proteins [98]. Moreover, the mitochondrial localization of both proteins might indicate that inflammation and fibrosis processes are regulated by mtROS production. Following the latter, in DN, hyperglycemia triggers the Smad4 translocation into mitochondria. This translocation reduces oxidative phosphorylation (OXPHOS), inducing inflammation, fibrosis, and podocyte injury [98].

The production of mtROS and ROS produced by NOXs also damages phospholipids and mitochondrial DNA (mtDNA). mtDNA is particularly susceptible to ROS, because it does not contain histones to protect it, causing DNA integrity loss and resulting in the acquisition of mutations [99]. mtROS also favor the opening of mitochondrial permeability transition pores (MPTPs) into the cytosol [86]. On the other hand, mtROS induce phospholipids oxidation, principally cardiolipin, leading to the ↓ΔΨm, MPTP opening, and ETS activity reduction [100]. Thus, the pathological crosstalk between NOXs and mitochondrial induces ROS, affecting mitochondrial metabolism and biomolecules integrity. The latter induces mitochondrial impairment, favoring the AKI to CKD transition.

## 5. Mitochondrial Metabolism, ROS, and OS in Kidney Diseases

The kidneys remove waste from the blood, reabsorb nutrients, regulate electrolyte balance, maintain acid-base homeostasis and regulate blood pressure [10]. These processes require high amounts of energy, which come from OXPHOS or anaerobic glycolysis, depending on the region of the kidneys. For example, the renal cortex uses OXPHOS and low amounts of glucose, while the renal medulla uses glycolysis and lactate. Therefore, the medulla necessarily uses anaerobic glycolysis due to low oxygen levels. In contrast, the renal cortex uses OXPHOS, fed mainly by FA oxidation [10,101].

**Figure 3:**
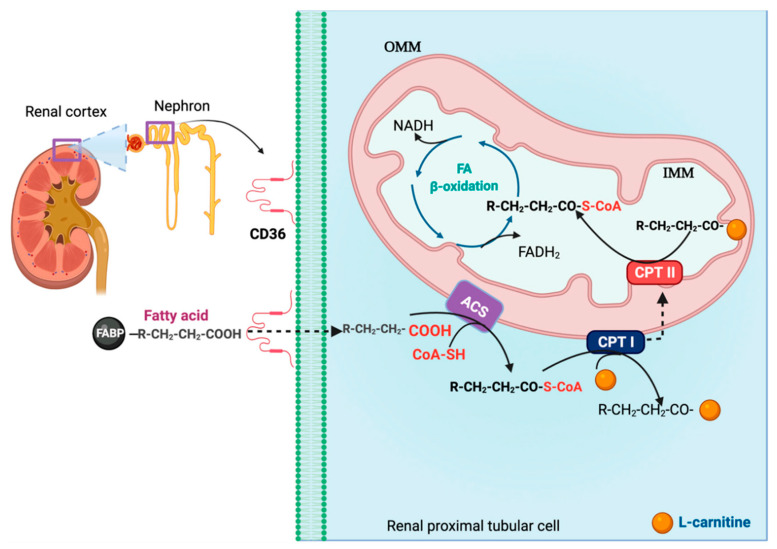
Renal proximal tubule (RPTC) cells use fatty acids (FA) β-oxidation to produce adenosine triphosphate (ATP). In the kidney, the proximal tubules of the nephrons of the renal cortex use fatty acids (FA) as the primary source of energy. FA bound to the fatty acid-binding protein (FABP) enter the RPTC through the cluster of differentiation 36 (CD36). In the cytosol, acyl-coenzyme A (CoA) synthetase (ACS) (attached to the outer mitochondrial membrane (OMM)) activates FA by the addition of acetyl-CoA (CoA-SH). The latter allows FA to enter the OMM through carnitine *O*-palmitoyl transferase I (CPT I). CPTI exchanges acetyl-CoA for L-carnitine. In turn, FA goes to the inner mitochondrial membrane (IMM). In the IMM, carnitine *O*-palmitoyl transferase II (CPT II) removes the carnitine group and adds acetyl-CoA (CoA-SH). The latter allows FA to enter the mitochondrial matrix. Fatty acyl-CoA undergoes β-oxidation, generating nicotinamide adenine dinucleotide phosphate (NADH) and flavin adenine dinucleotide (FADH_2_). Created with Biorender.com.

A significant number of mitochondria in renal cells is located in PT, the most metabolically active [10]. OXPHOS is the principal mechanism to produce ATP in renal proximal tubular cells (RPTCs). Ninety percentage of ATP is required to reabsorption of glucose, ions, and nutrients through the sodium-potassium ATP pump (Na^+^/K^+^ ATPase) [102]. PT uses FA, such as palmitate, through FA β-oxidation to produce high ATP levels (Figure 3) [103]. Therefore, RPTCs have high levels of carnitine *O*-palmitoyl transferase I (CPT I) isoforms A (CPT IA) and B (CPT IB), and carnitine *O*-palmitoyl transferase II (CPT II) [104].

### 5.1. FA β-Oxidation Dysfunction in Kidney Diseases

The impairment of β-oxidation has been reported in AKI and CKD. In patients and animal models, mRNAs along with β-oxidation proteins levels are decreased [105,106,107]. In the folic-acid-induced AKI model, ATP production is reduced, and mitochondria are uncoupling due to β-oxidation dysfunction [108]. Moreover, in maleic-acid (MA)-induced AKI, the FA β-oxidation-linked oxygen consumption rate (OCR) is diminished [109]. On the other hand, the transcriptomic analysis showed that the acyl-CoA dehydrogenase family member 10 (ACAD10) is downregulated in DN [110]. Moreover, in the 5/6 nephrectomy model, medium-chain acetyl dehydrogenase (MCAD) decreases at twenty-eight days [107]. Thus, the decrease in these enzymes is related to FA β-oxidation deregulation in AKI and CKD. Following the latter, FA β-oxidation has been evaluated in a course temporal in 5/6 nephrectomy, which decreases from early times [111]. Consequently, the reduction of FA β-oxidation causes intrarenal lipids accumulation, inducing lipotoxicity and impairing renal function [112]. In line with this, Nishi et al. [113] demonstrated that lipid accumulation is evident in tubular epithelial cells (TECs). Lipid deposition increases according to kidney lesion, suggesting a metabolic reprogramming that shifts β-oxidation to lipid synthesis [114,115]. According to the latter, UUO increases triglycerides synthesis from one day after obstruction [116]. Triglycerides increase is partly due to the overexpression of the transporter of the long-chain FA cluster of differentiation 36 (CD36) in PT [7,117]. CD36 also promotes signaling pathway activation such as epithelial–mesenchymal transition (EMT), inflammation, and others, leading to fibrosis. In this context, CD36^−/−^ mice subjected to UUO show less fibrosis than sham groups [118]. However, Kang et al. [106] showed that mice that overexpress CD36 show the accumulation of lipids but low expression levels of fibrotic markers. Therefore, the authors hypothesized that impaired FA β-oxidation is sufficient to induce the development of fibrosis and lipid accumulation is the only consequence of this dysfunction [106]. Thus, it has been suggested that defective FA β-oxidation is one of the principal mechanisms associated with fibrosis development [103,106].

The alterations in CPT I levels also contribute to FA β-oxidation impairment. For example, modifications in transporters of plasma acylcarnitine have been reported in CKD [119]. In this context, Prieto-Carrasco et al. [120] showed CPT I levels decreased in a temporal course from 2 to 28 days after nephrectomy, associated with progressive impairment in mitochondrial β-oxidation. The authors concluded that the decrease in CPT I favors mitochondrial β-oxidation impairment and the subsequence fibrosis development. The latter is supported by the fact that patients with CKD show a correlation between low CPT IA levels and fibrosis [121]. In this sense, the transgenic mouse models overexpressing CPT IA are able to restore oxidative metabolism, avoiding fibrosis development in UUO, folic acid nephropathy, and adenine-induced nephrotoxicity [121]. In addition, CPT IA overexpression also reduces fibrosis by decreasing TGFβ1 levels [121].

In summary, defective FA β-oxidation is observed in kidney diseases from early times, promoted through decreased mRNA expression and downregulation in the activity and levels of the proteins involved in this process and ETS activity reduction (discussed below). Later, the overexpression of CD36 contributes to lipid accumulation and the activation of mechanisms that lead to the fibrotic process. However, other factors contribute to the impairment of β-oxidation.

#### Oxidation and OS Production and in Kidney Diseases

Renal pathologies cause a disturbance in mitochondria homeostasis, affecting mitochondrial metabolism, which leads to AKI to CKD transition. OS might cause these alterations in mitochondrial metabolism produced during ETS, β-oxidation, and Krebs cycle activity [103]. For instance, Kowaltowski’s group [122,123] demonstrated that H_2_O_2_ is produced during the first step of FA β-oxidation, catalyzed by a very long-chain acyl-CoA dehydrogenase (VLCAD) enzyme in liver mitochondria. If H_2_O_2_ produced is not degraded, it could induce mitochondria-decoupling electron leakage during OXPHOS. Even β-oxidation may be damaged. The latter has been supported by Aparicio-Trejo et al. [44], showing that folic acid causes damage to mitochondria and decreasing the OXPHOS associated with FA β-oxidation is due to ROS overproduction. The use of the antioxidant N-acetylcysteine (NAC) prevents the reduction in OXPHOS capacity associated with FA β-oxidation impairment from 2 to 28 days of the administration, avoiding CKD transition [108]. In accordance, Briones-Herrera et al. [109] showed that MA, another inductor of OS, decreases β-oxidation and the use of antioxidant sulforaphane (SF) prevents this decrease [109].

On the other hand, Tan et al. [110] showed by transcriptomic analysis in diabetic mice that mitochondrial FA β-oxidation is downregulated. This downregulation is attributed to the overexpression of the C5 substrate of the complement system receptor 1 (C5aR1). Thus, C5aR1 is implicated in lipids metabolism in diabetes [124]. C5aR1 is also upregulated in kidney diseases, producing FA β-oxidation impairment in DN [110]. In addition, C5aR1 upregulation disrupts mitochondrial respiration, generating high levels of ROS. These results showed that C5aR1-induced ROS overproduction alters FA metabolism in DN.

**Figure 4:**
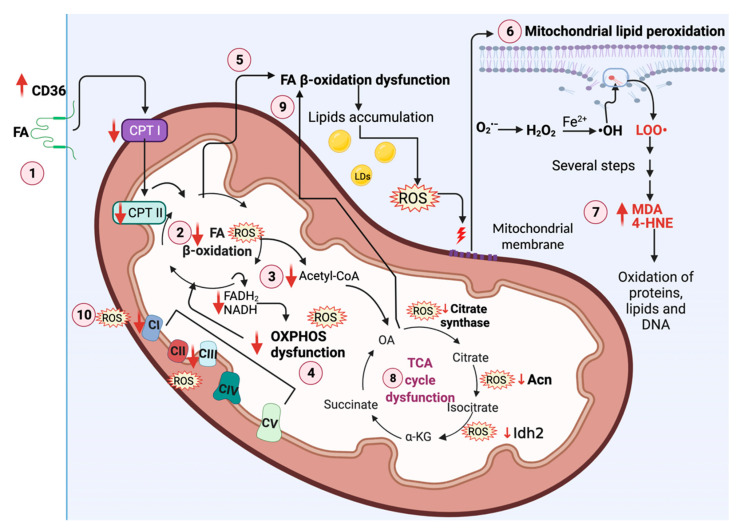
ROS deregulate mitochondrial metabolism in kidney diseases. (1) In renal damage, the cluster of differentiation 36 (CD36) overexpression causes a high fatty acids (FA) uptake. In addition, carnitine *O*-palmitoyl transferase I (CPT I) and carnitine *O*-palmitoyl transferase II (CPT II) are decreased. (2) ROS cause FA β-oxidation decrease, inducing (3) a tricarboxylic acid (TCA) cycle and (4) oxidative phosphorylation (OXPHOS) capacity reduction. The decrease in β-oxidation also (5) induces the accumulation of lipids and a further ROS overproduction. The latter (6) damages mitochondrial membranes by inducing mitochondrial lipid peroxidation, (7) forming the products malondialdehyde (MDA) and 4-hydroxynonenal (4-HNE). These products are highly reactive and damage other lipids, proteins, and mitochondrial DNA (mtDNA). On the other hand, (8) ROS downregulate aconitase (Acn), citrate synthase, and isocitrate dehydrogenase isoform 2 (Idh2), inducing TCA cycle dysfunction. Moreover, (9) TCA cycle impairment induces FA β-oxidation dysfunction. (10) ROS decrease CI and CIII activities, inducing β-oxidation dysfunction [111]. O_2_^•−^, superoxide anion radical; H_2_O_2_, hydrogen peroxide; ^•^OH, hydroxyl radical; LOO^•^, lipid peroxyl radical; LDs, lipid droplets; OA, oxalacetate; α-KG, alpha-ketoglutarate; DNA, deoxyribonucleic acid; NADH, nicotinamide adenine dinucleotide phosphate; FADH_2_, flavin adenine dinucleotide; CoA, coenzyme A; CII, complex II; CIV, complex IV; CV, complex V. Created with BioRender.com.

During mitochondria decoupling, electron leakage from ETS occurs, which induces the reduction of oxygen (O_2_) to the radical O_2_^•−^, a type of ROS that triggers the production of other ROS such as H_2_O_2_ [125]. High levels of ^•^OH and ONOO^−^ induce OS and significantly oxidative damage of proteins, lipids, and DNA. The oxidation of lipids produces highly reactive molecules such as MDA and 4-HNE as products of chain lipid peroxidation that also induce mtDNA damage (Figure 4) [68]. Forty-eight hours after cisplatin treatment, induced AKI, 4-HNE, and MDA levels increase along with GPx4 levels decrease in the renal cortex, indicating lipid membrane peroxidation [126]. Furthermore, in MA-induced Fanconi syndrome, 24 h after injection with MA, the mitochondrial levels of 4-HNE are elevated [109]. Additionally, GPx activity decreases, favoring H_2_O_2_ accumulation and mitochondrial lipidic peroxidation [109]. In folic-acid-induced AKI, 24 h after folic acid administration, mitochondrial MDA and 4-HNE levels increase [44]. Both OS markers also increase in nephrectomy models [127,128]. Together, these results show that mitochondria suffer lipid peroxidation in AKI and AKI to CKD transition.

In kidney diseases, the uptake of lipids by CD36, along with the dysfunction of FA β-oxidation, causes lipids accumulation in lipid droplets (LDs), inducing ROS overproduction (Figure 4) [129,130]. Since ROS and their products induce severe cell damage, a cellular balance of ROS is needed. This balance is performed by different antioxidants that include enzymatic and non-enzymatic antioxidants [131]. The reduction of the antioxidant system has been widely reported in kidney diseases [11,132]. In renal ischemia and nephrotoxicity, catalase (CAT), SOD, and glutathione S-transferase (GST) levels are depleted [70,133]. Moreover, in cisplatin-induced AKI, mitochondrial GSH and NADPH levels are decreased [134]. In the 5/6 nephrectomy, the activities of CAT, SOD, GPx, GR, and GST fall at 20 h in glomeruli, PT, and DT [128]. Moreover, early after UUO, these enzymes’ mRNA and protein levels decrease, while oxidative markers increase [135]. Indeed, the decrease in antioxidant-system-induced OS has been suggested as a factor to induce the AKI to CKD transition.

The decreases in acetyl-CoA induced by FA β-oxidation impairment reduce the TCA cycle capacity. Interestingly, in AKI and CKD models, the reduction in TCA cycle enzymes is observed, before FA β-oxidation dysfunction occurs, suggesting this point as the start of the vicious cycle, which further increases the mitochondrial damage (Figure 4). In the next section, we will address the impact that ROS have on TCA cycle dysfunction.

### 5.2. TCA Cycle Redox-Sensitive Signaling Pathway in Kidney Diseases

The urinary excretion of the non-diabetic CKD patients shows low levels of TCA cycle metabolites (e.g., citrate, cis-aconitate, isocitrate, alpha-ketoglutarate (α-KG), and succinate) [136]. In addition, kidney biopsies have reduced aconitate, isocitrate, alpha-ketoglutarate dehydrogenase (α-KGDH), and succinate gene expression. These results show TCA cycle dysfunction [136]. In contrast, in a mouse model of DN, pyruvate, citrate, α-KGDH, and fumarate are upregulated [137]. Moreover, in UUO, succinate levels increase, attributed to TCA cycle dysfunction [138]. Note that the amount of the metabolites is tissue- and disease-dependent, so the identification of these metabolites could give advantages in the early detection of mitochondrial damage in these diseases.

TCA cycle dysfunction might be attributed to ROS alterations. In vitro studies have postulated that high glucose oxidation rates lead to the excessive production of electron donors from the TCA cycle. As a consequence, ETS becomes overloaded, promoting O_2_^•^^−^ overproduction [139]. In line with this, podocytes treated with high glucose levels have high ROS levels, and the treatment with mitoTEMPO decreases them [140], suggesting that ROS are specifically delivered from mitochondria. Controversially, the determination of mtROS in the diabetic mouse model shows that it is reduced [141]. Further studies in vivo are needed to elucidate the mtROS overproduction-induced TCA cycle dysfunction in DN.

In the kidney, TCA cycle enzymes can be sulfenylated or S-glutathionylated. For example, Acn can be reversibly inactivated by the oxidation of the sulfhydryl group by O_2_^•^^−^ and H_2_O_2_. However, if OS persists, Acn can be irreversibly deactivated by the disruption of the 4Fe-4S group [142]. In AKI induced by folic acid, mitochondrial Acn activity decreases, and the pre-treatment with NAC prevents it [44], suggesting that ROS promote the deactivation of Acn. In addition, the relation between Acn and citrate synthase diminishes, supporting the idea that decreasing in Acn activity is related to OS [44]. Moreover, Mapuskar et al. [143] reported that the persistent increase of O_2_^•−^ decreases Acn and citrate synthase activity in cisplatin-induced kidney injury, of which the effects are ameliorated by SOD mimetic avasopasem manganese (GC4419) treatment. The authors reported that in the AKI phase, Acn and citrate synthase activities do not show changes, suggesting that high levels of ROS are required for their inactivation in this model [143]. The latter is demonstrated due to the fact that high levels of ROS are more evident in cisplatin-induced CKD [143].

In kidney pathologies, the levels of mitochondrial isocitrate dehydrogenase isoform 2 (Idh2) are decreased [144,145]. In the cisplatin model, Idh2 function is affected by decreased mitochondrial NADPH and GSH and increased H_2_O_2_ production [145]. Furthermore, Han et al. [144] showed that OS generated during I/R reduces Idh2 levels in kidney tubule cells from mice. Since S-glutathionylation deactivates Idh2, this Ox-PTM may be produced during OS under I/R conditions [146]. The deletion of the Idh2 (Idh2^−/−^) gene in these mice exacerbates kidney tubule injury by increasing plasma creatinine and blood urea nitrogen (BUN) levels. In addition, OS increases the reduction of mitochondrial NADP^+^ along with GST and GPx activities. In contrast, mitochondrial GSSG/GSH ratio augments. Idh2^−/−^ mice show mitochondrial dysfunction and fragmentation, which induces apoptosis in kidney tubule cells [144]. After UUO, Idh2 decreases, and its deletion increases OS markers such as 4-HNE and H_2_O_2_ in mitochondrial fractions [147]. Additionally, inflammatory cell filtration was more evident in Idh2^−/−^ than wild-type (WT) groups. Together, these results highlighted the importance of Idh2 in managing OS, and its deactivation exacerbates mitochondrial damage.

Note that the fact that ROS-induced TCA cycle dysfunction affects FA β-oxidation has been demonstrated, because TCA cycle impairment is observed early before FA β-oxidation damage in time course studies of the AKI to CKD transition, (Figure 4). In this regard, OXPHOS capacity is also decreased by ROS in early times, suggesting that both events are required to enhancement FA β-oxidation dysfunction [44,108,120].

### 5.3. OXPHOS Redox-Sensitive Signaling Pathway in Kidney Diseases

As mentioned above, kidney energy demand depends on OXPHOS, which in turn is regulated by Ox-PTMs. However, in the context of kidney diseases, it is poorly studied. OXPHOS capacity is downregulated in renal diseases, inducing ROS overproduction. In this regard, the production of mitochondrial H_2_O_2_ has been reported in kidney injury models, associated with ↓ΔΨm, decreasing OXPHOS capacity [8,148]. In remnant kidney from 5/6 nephrectomy, OXPHOS linked to complex I (CI) feeding decrease in a temporal course of nephrectomy from 2 to 28 days [111]. Moreover, male Sprague Dawley rats subjected to nephrectomy showed ATPβ, NDUSF8, and cytochrome c oxidase subunit 1 (Cox I) reduction [8]. Consequently, CI, complex III (CIII) activities, and cyt c diminished, impairing mitochondrial function [8]. Avila-Rojas et al. [148] reported that potassium dichromate (K_2_Cr_2_O_7_) decreases the CI + CII-linked S3 respiratory state. In addition, ΔΨm in CI + complex II (CII)-linked respiration and respiration associated with OXPHOS are reduced, suggesting that K_2_Cr_2_O_7_ principally affects the synthesis of mitochondrial ATP [148]. Although redox signaling has not been investigated in previous studies, components of OXPHOS might be regulated by Ox-PTMs.

## 6. ROS Induce Uncoupling Proteins (UCPs) Dysregulation in Kidney Diseases

UCPs are proton transporters (H^+^), which move H^+^ from the IMM into the mitochondrial matrix. These transporters are localized in the IMM and dissipate the proton gradient from the mitochondrial matrix into the IMS [149]. mtROS induce UCP2 activation, decreasing the proton gradient and preventing mtROS overproduction [149].

It has been shown that UCP2 deletion aggravates tubular injury in the I/R model by inducing ROS overproduction, supporting the importance of these transporters in ROS dissipation [150]. Moreover, the UCP2 inhibition worsens the damage caused by lipopolysaccharide (LPS), increasing apoptosis in TECs [151]. In CKD, Jian et al. [152] showed that in renal tubular cells (RTCs), the expression of UCP2 is induced three days after obstruction and continues after seven days, avoiding UUO-induced fibrosis. It suggests that UCP2 is crucial to avert fibrosis development induced by ROS in UUO.

Although UCP1 is commonly found in mitochondria from brown adipose tissue, it is expressed in the kidney. For instance, in AKI models induced by cisplatin or I/R, Jia et al. [153] found that UCP1 is upregulated in renal TECs and its presence is related to OS suppression. Chouchani et al. [154] showed that mtROS alter the redox status of UCP1 by inducing its sulfenylation in Cys 253, promoting UCP1 activity.

## 7. Redox-Sensitive Signaling Controls Mitochondrial Dynamics, Biogenesis, and Mitophagy

Mitochondrial dynamic is the balance between mitochondrial fusion and fission, and it is involved in regulating mitochondrial metabolism and cell death. Likewise, the shape and morphology of mitochondria are regulated by the metabolite and ROS concentration concentrations [155]. The integral membrane guanosine triphosphatases (GTPases) perform mitochondrial fusion: mitofusin 1 (Mfn1), mitofusin 2 (Mfn2), and optical atrophy 1 (Opa1) [136]. Meanwhile, mitochondrial fission is mediated by Drp1, the mitochondrial fission factor (Mff), the adaptor protein fission protein 1 (Fis1), and the mitochondrial elongation factor 1 (Mief1) and 2 (Mief2). Chronic OS has been shown to induce mitochondrial fission [155]. However, H_2_O_2_ sublethal amounts, and acute OS cause mitochondrial hyperfusion [156]. In HeLa cells, mitochondrial hyperfusion has been associated with the S-glutathionylation of Mfn2 and the formation of Opa1 oligomers. Moreover, mitochondrial hyperfusion increases the resistance to cellular stress and cell death, since it promotes antioxidant defense enzymes activation [156]. In the next section, we analyze the current trends and paradigms of ROS-mediated signaling that form a link between redox-sensing elements and mitochondrial dynamics and their possible role in kidney diseases.

### 7.1. Redox-Sensitive Proteins Participating in Fission and Fusion

Redox signaling conveys external and internal signals between redox-sensitive receptors and the downstream effectors of fission machinery. Mitochondrial dynamics require the recruitment of proteins to mitochondria. Indeed, the importation of several proteins to mitochondria depends on proton electrochemical gradient H^+^ created by ETS at the IMM, which is called the proton motive force (PMF) [157]. In addition, several redox-sensitive proteins are activated to induce proteins translocation from the cytosol. For instance, previous studies in HeLa cells have shown that MAPKs are involved in regulating the fission process in response to OS through Ras [158,159]. In line with this, the redox-sensitive extracellular regulated kinase 2 (ERK2) protein mediates the phosphorylation of Drp1 in Ser 616 to induce mitochondrial fragmentation [158]. In addition to ERK 2, PKC-delta (PKC-δ) promotes Drp1 phosphorylation in Ser 579 under OS [160]. Drp1 is also regulated by ROS. In this context, Kim et al. [161] showed that in vascular diseases related to diabetes and aging models, protein disulfide isomerase A1 (PDIA1) is depleted, inducing the sulfenylation of Drp1 in Cys 644. The latter leads to mitochondrial fragmentation, mtROS increase, and senescence induction. Furthermore, the authors demonstrated that the restoration of the PDIA1/Drp1 axis could be used as a therapeutic strategy to improve vascular diseases [161], suggesting that PDIA1 has a thiol reductase function for Drp1. Drp1 persulfidation was previously reported, inducing its inactivation. Persulfidation is carried out by CARS2, altering mitochondrial dynamics and favoring fusion [66].

**Figure 5:**
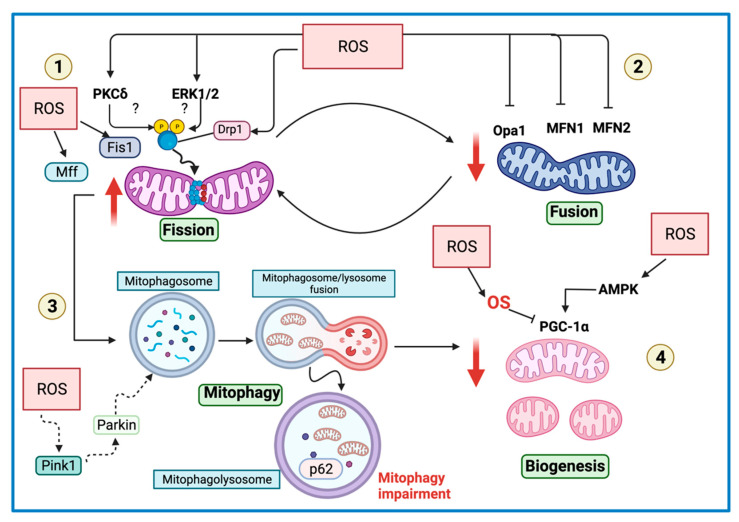
Redox-sensitive signaling regulates mitochondrial dynamics in kidney diseases. (1) ROS activate the redox-sensitive extracellular regulated kinase 2 (ERK2) protein and protein kinase C (PKC) isoform δ (PKC-δ). These proteins phosphorylate and activate dynamin-related protein 1 (Drp1), inducing its translocation to the OMM to triggering fission. Likewise, ROS overproduction upregulates Drp1, fission 1 (Fis1), and mitochondrial fission factor (Mff), inducing fission increase. (2) ROS also induce fusion decrease by downregulating optical atrophy 1 (Opa1) and mitofusin 1 (Mfn1) and 2 (Mfn2) proteins. (3) The augment of fission promotes mitophagy activation by inducing the translocation of phosphatase and tensin homolog (PTEN)-induced putative kinase 1 (Pink1) that in turn recruit to parkin in the OMM to induce mitophagosome formation. Mitophagosome fuses with lysosome to form mitophagolysome formation. However, mitophagy flux is impaired, inducing the accumulation of damaged mitochondria. (4) Low ROS levels induce mitochondrial biogenesis by activating the redox-sensitive adenosine monophosphate (AMP)-activated protein kinase (AMPK) and the peroxisome proliferator-activated receptor (PPAR) γ coactivator-1 alpha (PGC-1α). However, high levels of ROS producing oxidative stress (OS) induce mitochondrial biogenesis decrease, triggering mitochondrial mass decrease. Created with BioRender.com.

In renal pathologies, the upregulation of Drp1 is related to OS conditions [44,147,148]. Likewise, the phosphorylation of ERK 1/2 increases along with Drp1 levels in response to OS in the I/R rat model [162]. Thus, ROS might induce the recruitment of Drp1 through ERK1/2 (Figure 5).

In kidney diseases, ROS overproduction induces mitochondrial proteins fusion decrease and fission increase. In line with this, in the K_2_Cr_2_O_7_ rat model, Drp1 increase and curcumin treatment decrease it [148], suggesting that ROS induce Drp1 upregulation (Table 1). The latter is supported by the fact that ROS promote Drp-1 translocation to mitochondria. Drp1 is also recruited in RPTC treated with cisplatin, inducing mitochondrial fragmentation and ATP depletion [163]. Moreover, Drp1 is upregulated in Idh2^−/−^ mice due to NADPH and GSH decrease, which increase mtROS. mtROS also downregulate Opa1 (Table 1) [44,144,145]. Thus, mtROS trigger fission increase and fusion decrease. Following the latter, Opa1 levels are diminished under H_2_O_2_ treatment, and the diminishment is even higher in Idh2 small interfering RNA (siRNA)-transfected mProx24 cells [144]. Mfn1 is another fusion protein decreased in AKI models (Table 1) [44,164].

The blockage of mitochondrial fission has been suggested as a strategy to ameliorates mitochondrial damage [166]. For instance, the loss of DRP1, six hours after suffering bilateral I/R, re-establish mitochondrial function due to the reduction of mtROS production associated with fission decrease (Table 1) [165]. In addition, mice Drp1^−/−^ does not have tubulointerstitial fibrosis [165], suggesting that the Drp1 blocking might avoid the AKI to CKD transition. Furthermore, the specific inhibitor of Drp1, the mitochondrial division inhibitor 1 (mdivi-1), ameliorates I/R-mediated AKI (Table 1) [163]. However, in the case of UUO, the usage of mdivi-1 augments fibrosis [167], which correlates with midivi-1-treated human proximal tubular cells (HK2) under hypoxic conditions, showing fibrosis markers increase [167]. Therefore, Drp1 blocking might be employed in the case of AKI, but not in CKD.

The management of redox recovery homeostasis might be utilized as a strategy to improve mitochondrial dynamics. Consistent with this, the use of antioxidants that target mitochondria has been shown to enhance the homeostasis of mitochondrial dynamics [44,148]. Therefore, ROS regulate proteins involved in mitochondrial fission and fusion. According to the latter, it has been hypothesized that the treatment with sublethal amounts of H_2_O_2_ induces acute stress, promoting a hyperfused mitochondrial state [24,168]. In folic-acid-induced kidney injury, Drp1 and Fis1 increase, and the treatment with NAC decreases them [44]. In response to cisplatin or bilateral I/R, Drp1 is recruited to mitochondria, triggering apoptosis induction by delivering cyt c and decreasing the antiapoptotic protein B cell lymphoma 2 (Bcl-2) [163]. Moreover, the activation of peroxisome proliferator-activated receptor γ (PPARγ) stabilizes mitochondrial potential, reducing ROS. The latter results in decreasing Drp1, augmenting Mfn2, Opa1 and restoring mitochondrial dynamics [8], suggesting that the rescue of mitochondrial biogenesis might recover mitochondrial dynamics due to mtROS decrease. In CKD, the levels of fusion proteins (e.g., Mfn1, Mfn2, and Opa1) are also decreased, while fission proteins are increased (e.g., Drp1 and Mff) (Table 2) [8,167,169,170], attributed to mitochondrial OS increase.

Ox-PTMs might regulate mitochondrial dynamics proteins; however, they are poorly studied in kidney disease. In line with this, S-nitrosylation in Drp1 Ser 644 induces Drp1 dimerization and augments its GTPase activity. Thus, NO promotes Drp1-induced mitochondrial fission [171]. Furthermore, OS can trigger mitochondrial hyperfusion [156]. Further studies are needed to clarify the role of ROS to induce Ox-PTMs regulation in mitochondrial dynamics and biogenesis in renal disease.

### 7.2. Redox-Sensitive Proteins Participating in Mitochondrial Biogenesis

Mitochondria biogenesis is triggered to increase the number and size of mitochondria [172]. PPARγ coactivator-1 alpha (PGC-1α) controls the biogenesis process through the transcription of nuclear respiratory factors 1 (NRF-1) and 2 (NRF-2), PPARs, transcription factor A (TFAM), estrogen, and estrogen-related receptors (ERRs), among others [173]. These proteins are redox-sensitive. For instance, the exposure to H_2_O_2_ in skeletal muscle cells for 24 h increases the activity of the PGC-1α promoter as well as mRNA expression. These effects are blocked with NAC treatment [174], showing that ROS mediate the activation of PGC-1α. However, the impact of ROS over PGC-1α depends on the concentration and cellular type. For example, low ROS levels lead to the reduced expression of PGC-1α, while high levels induce its transcription through redox-sensitive adenosine monophosphate (AMP)-activated protein kinase (AMPK) [174], which function as a cellular energy sensor by regulating mitochondrial biogenesis and maintaining redox homeostasis [175]. AMPK reduces ROS through PGC1-α, which induces the overexpression of CAT, Mn-SOD, UCP2, and nicotinamide adenine dinucleotide (NAD)-dependent deacetylase sirtuin-3 (SIRT3) [176]. Therefore, the activation of AMPK induces PGC-1α promoter activity and mRNA levels increase. PGC-1α is commonly downregulated in kidney diseases, leading to mitochondrial mass and metabolism decrease [10]. In the folic-acid-induced AKI model, PGC-1α, TFAM, NRF1, and NRF2 levels decrease 24 h after the treatment, and NAC treatment prevents this effect [44]. Moreover, male Wistar rats subject to nephrotoxicity by K_2_Cr_2_O_7_ show a decrease in PGC-1α levels [148]. Both studies showed that the treatment with antioxidants (NAC and curcumin) upregulates biogenesis [47,152,169]. Importantly, NAC and curcumin antioxidants have shown mitochondria protection by promoting bioenergetics preservation and maintaining redox homeostasis to avoid mtROS [16,94]. In 5/6 nephrectomy-induced CKD, the levels of PGC-1α decrease in a temporal course from two days after the nephrectomy, causing reductions in NRF1 and NRF2 [120]. The treatment with pioglitazone, an antidiabetic drug, reduces mtROS, restoring mitochondrial biogenesis. Interestingly, the biogenesis decrease in DN is attributed to the low production of O_2_^•−^ [141]. AMPK activity decreases in this model, and AMPK activation induces O_2_^•−^ production, activating PGC-1α [141]. Therefore, in the DN mice model, low ROS levels are essential factors to trigger mitochondrial biogenesis.

### 7.3. Mitophagy, ROS, and OS in Kidney Diseases

The dysregulation of mitophagy has been previously reported in renal diseases [177,178]. At biological levels, ROS are involved in mitophagy regulation. Upon mitochondrial damage, depolarization, or mitochondrial OS, mitophagy is triggered. ROS promote the recruitment of phosphatase and tensin homolog (PTEN)-induced putative kinase 1 (Pink1) in the outer mitochondrial membrane (OMM) [179]. The latter leads to the recruitment and phosphorylation of parkin to begin mitophagy [180]. In the AKI models, Pink1 and parkin are upregulated after damage [44,109,164]. The upregulation of these proteins is related to ROS and mtROS increase. Furthermore, in CKD models, both proteins are augmented [120,167,169]. For instance, in the 5/6 nephrectomy model, levels of Pink1, parkin, microtubule-associated protein 1A/1B-light chain 3 phosphatidylethanolamine conjugate (LC3-II), and sequestosome (p62) increase in a temporal course way [120]. However, in both cases, mitophagy has been reported impaired. Although the levels of LC3-II increase, the accumulation of p62 is evident, suggesting dysfunctional mitophagy [120]. p62 accumulation is considered a marker of mitophagy malfunction. In kidney injury, mitophagosome accumulation is evident by increasing Pink1, parkin, and p62 [181]. Together, these data suggest that ROS induce Pink1 and parkin translocation to mitochondria, but the high ROS levels exacerbate mitophagy machinery, damaging it. Mitophagy damage is supported, because antioxidants can increase mitophagy flux in AKI and CKD models [44,181].

On the other hand, excessive autophagy has been described in models of UUO, which triggers endothelial dysfunction [182]. Consistent with this, Chen et al. [183] found that treatment with NaHS, an exogenous H_2_S donor, in UUO mice decreases the expression levels of LC3-II/I, beclin-1, and AMPK proteins. On the contrary, the p62, CBS, and CSE levels increase compared to in the sham groups. Therefore, H_2_S is considered a protective mechanism, because it moderates OS that promotes the dysregulation of autophagy.

## 8. Concluding Remarks

ROS are second messengers that modify redox-sensitive proteins in mitochondria by inducing Ox-PTMs. Thus, low levels of ROS are necessary to render these modifications. However, in kidney diseases, ROS trigger mitochondrial dysfunction evident by alterations in FA β-oxidation, TCA cycle, OXPHOS, mitophagy, mitochondrial dynamics, and biogenesis.

Moreover, the crosstalk between NOXs and mitochondria generates ROS. The impairment in one of these elements can trigger an uncontrolled ROS production increase. Therefore, perturbations in mitochondrial redox homeostasis are common characteristics that allow the transition from AKI to CKD.

## Figures and Tables

**Table 1:** ROS regulate mitochondrial dynamics, biogenesis, and mitophagy in acute kidney injury (AKI) models.

AKI Model	In Vivo Model	Mitochondrial Dynamic Protein	Mechanism	References
Cisplatin-induced nephrotoxicity and I/R	C57BL/6 mice	↑Drp1	Drp1 translocates to the mitochondria in response to ROS overproduction.	Brooks et al. [163]
Maleate-induced nephrotoxicity	Male Wistar rats	↑Drp1, Fis1	Maleate-induced OS promotes mitochondrial fission by increasing Drp1 and Fis1.	Molina-Jijón et al. [84]
Cisplatin	C57BL/6 mice	↓Opa1, Mfn1 ↑Fis1 ↑Pink1, parkin	ROS and mtROS promote fission and decrease the mitochondrial fusion process.	Ortega-Domínguez et al. [164]
I/R	C57BL/6 mice	Drp1^−/−^	The deletion of Drp1 improves mitochondrial function by decreasing mtROS.	Perry et al. [165]
Cisplatin	Female C57BL/6 Idh2^−/−^ mice	↓Opa1 ↑Drp1	Idh2^−/−^ decreases NADPH and GSH levels, inducing OS and triggering fission increase and fusion decrease.	Kong et al. [145]
I/R	Female C57BL/6 Idh2^−/−^ mice	↑Drp1, Fis1 ↓Opa1	Idh2^−/−^-induced mtROS, decreasing the levels of fusion proteins and augmenting fission proteins.	Han et al. [144]
Folic acid	Male Wistar rats	↑Fis1, Drp1 ↓Opa1, Mfn1 ↑Pink1, ↓LC3 ↓PGC-1α, ↓NRF1, NRF2	ROS overproduction increases fission and reduces the fusion process.	Aparicio-Trejo et al. [44]
Nephrotoxicity by K_2_Cr_2_O_7_	Male Wistar rats	↑Drp1 ↓PGC-1α	ROS overproduction increases fission and reduces biogenesis.	Ávila-Rojas et al. [148]
MA: induced Fanconi syndrome	Male Wistar rats	↓TFAM, ↑Fis1, Drp1 ↑Parkin, p62, LC3-II	SF prevents mitochondrial fission increase and TFAM decrease and regulates mitophagy.	Briones-Herrera et al. [109]

Abbreviations: ↑: increase; ↓: decrease; I/R, ischemia/reperfusion; Drp1, dynamin-related protein 1; Fis1, fission 1; OS, oxidative stress; Opa1, optical atrophy 1; Mf1, mitofusin 1; Pink1, phosphatase and tensin homolog (PTEN)-induced putative kinase 1; Idh2, Isocitrate dehydrogenase isoform 2; K_2_Cr_2_O_7_, potassium dichromate; PGC-1α, peroxisome proliferator-activated receptor (PPAR) γ coactivator-1 alpha; TFAM, transcription factor A; NRF1, nuclear respiratory factor 1; NRF2, nuclear respiratory factor 2; p62, sequestosome; LC3, microtubule-associated protein 1A/1B-light chain 3 phosphatidylethanolamine conjugate; NADPH, nicotinamide adenine dinucleotide phosphate; GSH, glutathione; mtROS, mitochondrial reactive oxygen species; MA, maleic acid; SF, sulforaphane.

**Table 2:** ROS regulate mitochondrial dynamics, biogenesis, and mitophagy in chronic kidney disease (CKD) models.

CKD Model	In Vivo Model	Mitochondrial Dynamic Proteins Alteration	Effects	References
DN	Male C57BL/6J mice	↓PGC-1α, AMPK	Reduced ROS levels decrease mitochondrial biogenesis.	Dugan et al. [141]
5/6 nephrectomy	Male Sprague-Dawley rats	↓Mfn2, Opa1 ↑Drp1 ↓PPARγ	The use of pioglitazone, a peroxisome proliferator-activated receptor γ (PPARγ) activator, decreases mtROS, improving mitochondrial dynamics.	Sun et al. [8]
5/6 nephrectomy	Male Wistar rats	↑Mfn1, Opa1 ↓Fis1, Drp1	ROS overproduction favors mitochondrial fusion.	Aparicio-Trejo et al. [128]
UUO	Male C57BL/6J mice	↑LC3, Pink1, parkin	ROS-induced senescence impairs mitophagy.	Liu et al. [169]
UUO	Male C57BL/6J mice	↓Drp1 ↑LC3, Pink1, parkin	mtROS recruit Drp1 to the OMM, regulating mitophagy parkin-dependent.	Li et al. [167]
5/6 Nephrectomy	Male Wistar rats	↓NRF1, NRF2, TFAM PGC-1α, PPARα ↓Mfn2, Opa1 ↑LC3, p62	Mitochondrial biogenesis and dynamics are altered temporal courses.	Prieto-Carrasco et al. [111]

Abbreviations: ↑: increase; ↓: decrease; DN, diabetic nephropathy; PPARα, peroxisome proliferator-activated receptor γ coactivator-1α; AMPK, adenosine monophosphate (AMP)-activated protein kinase; PPARγ, peroxisome proliferator-activated receptor γ; OMM, outer mitochondrial membrane; Drp1, dynamin-related protein 1; Fis1, fission 1; OS, oxidative stress; Opa1, optical atrophy 1; Mf1, mitofusin 1; Pink1, phosphatase and tensin homolog (PTEN)-induced putative kinase 1; PGC-1α, peroxisome proliferator-activated receptor (PPAR) γ coactivator-1 alpha; TFAM, transcription factor A; NRF1, nuclear respiratory factor 1; NRF2, nuclear respiratory factor 2; p62, sequestosome; LC3, microtubule-associated protein 1A/1B-light chain 3 phosphatidylethanolamine conjugate.

## Data Availability

Not applicable.

## Abbreviations

↓ΔΨm	mitochondrial membrane potential depolarization
^•^NO	nitric oxide
^•^OH	hydroxyl radical
4-HNE	4-hydroxynonenal
α-KΓ	Alpha-ketoglutarate
α-KΓΔH	alpha-ketoglutarate dehydrogenase
ACAD10	acyl-CoA dehydrogenase family member 10
ACS	acyl-CoA synthetase
Acn	aconitase
AGE	advanced glycation end products
AKR1A1	aldo-keto reductase family 1 member A1
AKI	acute kidney injury
AMPK	adenosine monophosphate (AMP)-activated protein kinase
Ang II	angiotensin II
Arg	arginine
ATP	adenosine triphosphate
ATR-1	angiotensin type 1 receptor
Bcl-2	B cell lymphoma 2
BUN	blood urea nitrogen
CI	complex I
CII	complex II
CIII	complex III
CIV	complex IV
CV	complex V
C5aR1	C5 substrate of the complement system receptor 1
CAT	catalase
CBS	cystathionine β-synthase
CD36	cluster of differentiation-36
CKD	chronic kidney diseases
CoA	coenzyme A
Cox I	cytochrome c oxidase subunit 1
CPT I	carnitine *O*-palmitoyl transferase I
CPT II	carnitine *O*-palmitoyl transferase II
CPT IA	CPT I isoform A
CPT IB	CPT I isoform B
CSE	cystathionine γ-lyase
Cu/Zn-SOD	copper/zinc-SOD
Cys	cysteine
CARSs	cysteinyl-transfer RNA (tRNA) synthetases
Cyt c	cytochrome c
DKD	diabetic kidney disease
DN	diabetic nephropathy
DNA	deoxyribonucleic acid
Drp1	dynamin-related protein 1
DT	distal tubules
EGFR	epidermal growth factor receptor
EMT	epithelial–mesenchymal transition
eNOS	endothelial nitric oxide synthase
ERK2	extracellular regulated kinase 2
ERR	estrogen and estrogen-related receptors
ETS	electron transport system
FA	fatty acids
FABP	fatty-acid-binding protein
FADH_2_	flavin adenine dinucleotide
Fe^2+^	ferrous iron
Fe^3+^	ferric iron
Fe-S	iron-sulfur
Fis1	fission protein 1
FoxO	forkhead box O
GC4419	avasopasem manganese
GFRs	growth factor receptors
GPx	glutathione peroxidase
Grx	glutaredoxin
GSH	glutathione
GTPases	guanosine triphosphatases
GST	glutathione S-transferase
h	hours
HK2	human proximal tubular cells
HS^•^	hydrosulfide radical.
H_2_O_2_	hydrogen peroxide
H_2_S	gydrogen sulfide
His	histidine
I/R	ischemia/reperfusion
Idh2	isocitrate dehydrogenase isoform 2
IKKγ	regulatory subunit of inhibitor IKK complex
IMM	inner mitochondrial membrane
IMS	intermembrane space
K_2_Cr_2_O_7_	potassium dichromate
L-NAME	N(G)-nitro-L-arginine-methyl ester
LC3-II	microtubule-associated protein 1A/1B-light chain 3 phosphatidylethanolamine conjugate
LDs	lipid droplets
LOO^•^	lipid peroxyl radical
LPS	lipopolysaccharides
Lys	lysine
MA	maleic acid
MAPK	mitogen-activated protein kinases
MCAD	medium-chain acetyl dehydrogenase
MDA	malondialdehyde
Met	methionine
MetO (R–SOCH_3_)	methionine sulfoxide
MetO_2_ (RSO_2_CH_3_)	methionine sulfone
Mff	mitochondrial fission factor
Mfn1	mitofusin 1
Mfn2	mitofusin 2
Mief1	mitochondrial elongation factor 1
Mief2	mitochondrial elongation factor 2
mitoTEMPO	2-(2,2,6,6-tetramethylpiperidin-1-oxyl-4-ylamino)-2-oxoethyl) triphenylphosphonium chloride
mitoQ	mitochondrial-targeting antioxidants coenzyme Q
Mn-SOD	manganese superoxide dismutase
mRNA	messenger RNA
Msr	methionine sulfoxide reductase
MsrA	Msr isoform A
MPTP	mitochondrial permeability transition pore
mt-K_ATP_	mitochondrial ATP-sensitive potassium K channel
mtDNA	mitochondrial DNA
mtROS	mitochondrial ROS
Na^+^/K^+^ ATPase	sodium–potassium ATP pump
NAC	N-acetylcysteine
NAD	nicotinamide adenine dinucleotide
NADPH	nicotinamide adenine dinucleotide phosphate
NaHS	sodium hydrosulfide
NF-κB	nuclear factor kappa-light-chain-enhancer of activated B cells
NOS	nitric oxide synthase
NOXs	NADPH oxidases
NRF-1	nuclear respiratory factor 1
NRF-2	nuclear respiratory factor 2
Nrf2	nuclear factor erythroid 2–related factor 2
O_2_	oxygen
O_2_^•−^	superoxide anion radical
OA	oxalacetate
OCR	oxygen consumption rate
OMM	outer mitochondrial membrane
ONOO^−^	peroxynitrite
Opa1	optical atrophy 1
OS	oxidative stress
Ox-PTMs	oxidative post-translational modifications
OXPHOS	oxidative phosphorylation
p62	sequestosome
PDIA1	protein disulfide isomerase A1
PGC-1α	peroxisome proliferator-activated receptor (PPAR) γ coactivator-1 alpha
Pink1	phosphatase and tensin homolog (PTEN)-induced putative kinase 1
PKC	protein kinase C
PKC-ε	PKC-epsilon
PKC-δ	PKC-delta
PKM2	pyruvate kinase isoform M2
PMF	proton motive force
PPARs	peroxisome proliferator-activated receptor
Pro	proline
Prx	peroxiredoxin
PT	proximal tubules
PTKs	protein tyrosine kinases
PTM	post-translational modifications
PTPs	protein tyrosine phosphatases
R–S^•^	thyil radical
R–SNO	S-nitrosothiol
R–SOH	sulfenic acid
R–SO_2_H	sulfinic acid
R–SO_3_H	sulfonic acid
R–S(O)–S–R	thiosulfinate
R–SN–R’	sulfenamide
R–S–S–H	persulfonation
Rac1	Ras-related C3 botulinum toxin substrate 1
RAGE	AGE receptor
ROS	reactive oxygen species
RPTCs	renal proximal tubular cells
RTC	renal tubular cells
S^−^	thiolate anion
S–S	disulfide bonds
SF	sulforaphane
SH	thiol
siRNA	small interfering RNA
SIRT3	nicotinamide adenine dinucleotide (NAD)-dependent deacetylase sirtuin-3
S_N_2	bimolecular nucleophilic substitution
SSG	glutathionylation
TCA	tricarboxylic acid
TECs	tubular epithelial cells
TFAM	transcription factor A
TGFβ1	transforming growth factor-beta 1
Thr	threonine
Trx	thioredoxin
Tyr	tyrosine
UCPs	uncoupling proteins
UCP1	uncoupling protein 1
UCP2	uncoupling protein 2
UCPs	uncoupling proteins
UUO	unilateral ureteral obstruction
VLCAD	very long-chain acyl-CoA dehydrogenase
WT	wild type
